# Supplementary material for: Polycomb repressive complex 2 structure with inhibitor reveals a mechanism of activation and drug resistance
Source: Nat Commun. 2016 Apr 28;7:11384. doi: 10.1038/ncomms11384 (PMC4853478; doi:10.1038/ncomms11384)
Supplement: Supplementary Information — Supplementary Figures 1-9, Supplementary Table 1, Supplementary Methods and Supplementary References. [file ncomms11384-s1.pdf]

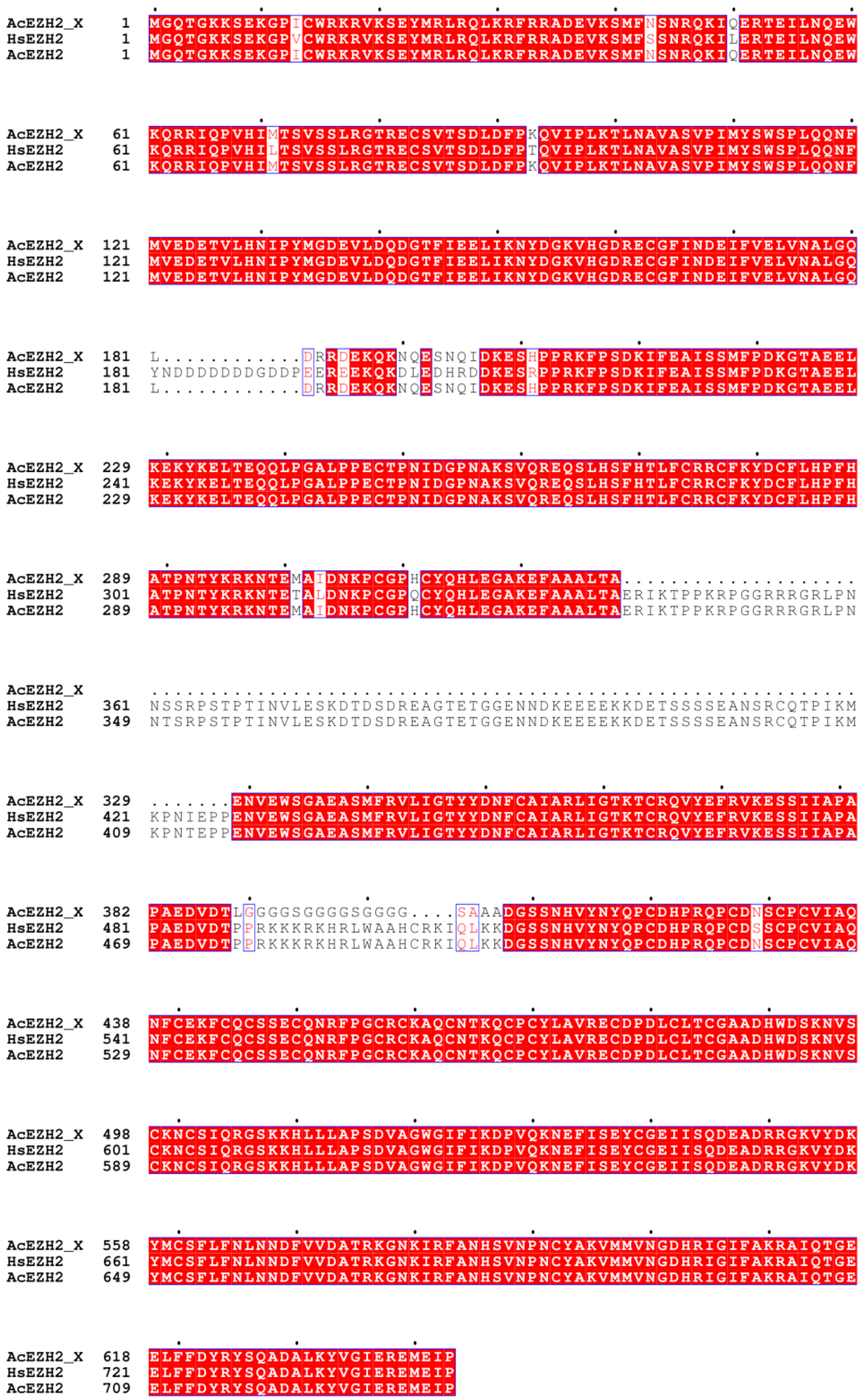

Supplementary Figure 1. Primary protein sequence alignment of engineered *Ac*EZH2_ X, *Hs*EZH2 (Q15910) and *Ac*EZH2 (G1KPH4)[1]. Sequence alignment was performed using BLAST.

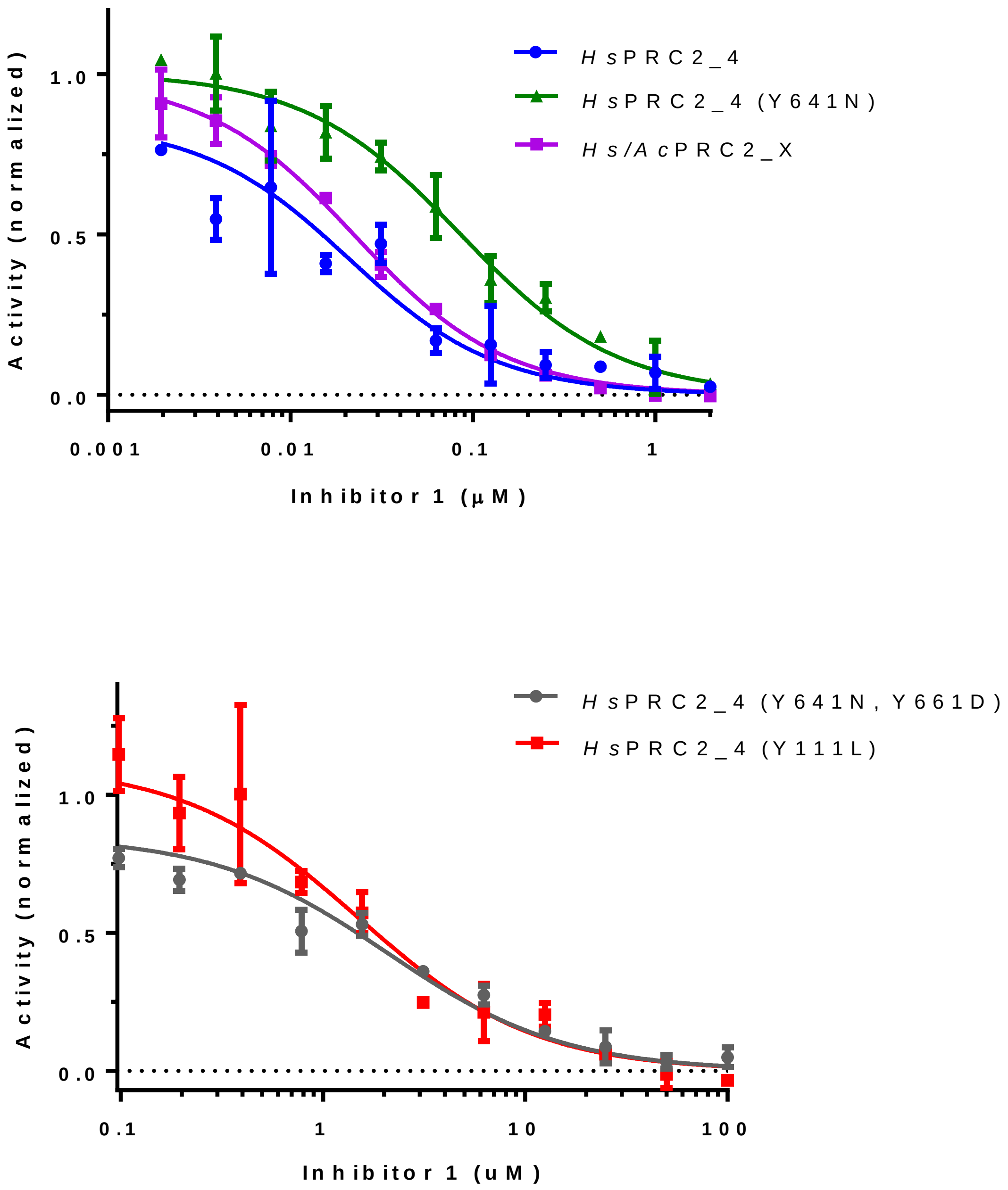

Supplementary Figure 2. Inhibition of intact *Hs*PRC2_4 protein complexes (WT, Y111L, Y641N and Y641N/Y661D) and *Hs/Ac*PRC2_X by inhibitor 1. All experiments were conducted at 50 µM SAM which is above $K_{m,}^{SAM}$ = 3-9 µM for various constructs (measurements are plotted as mean of two independent experiments ± s.d).

a

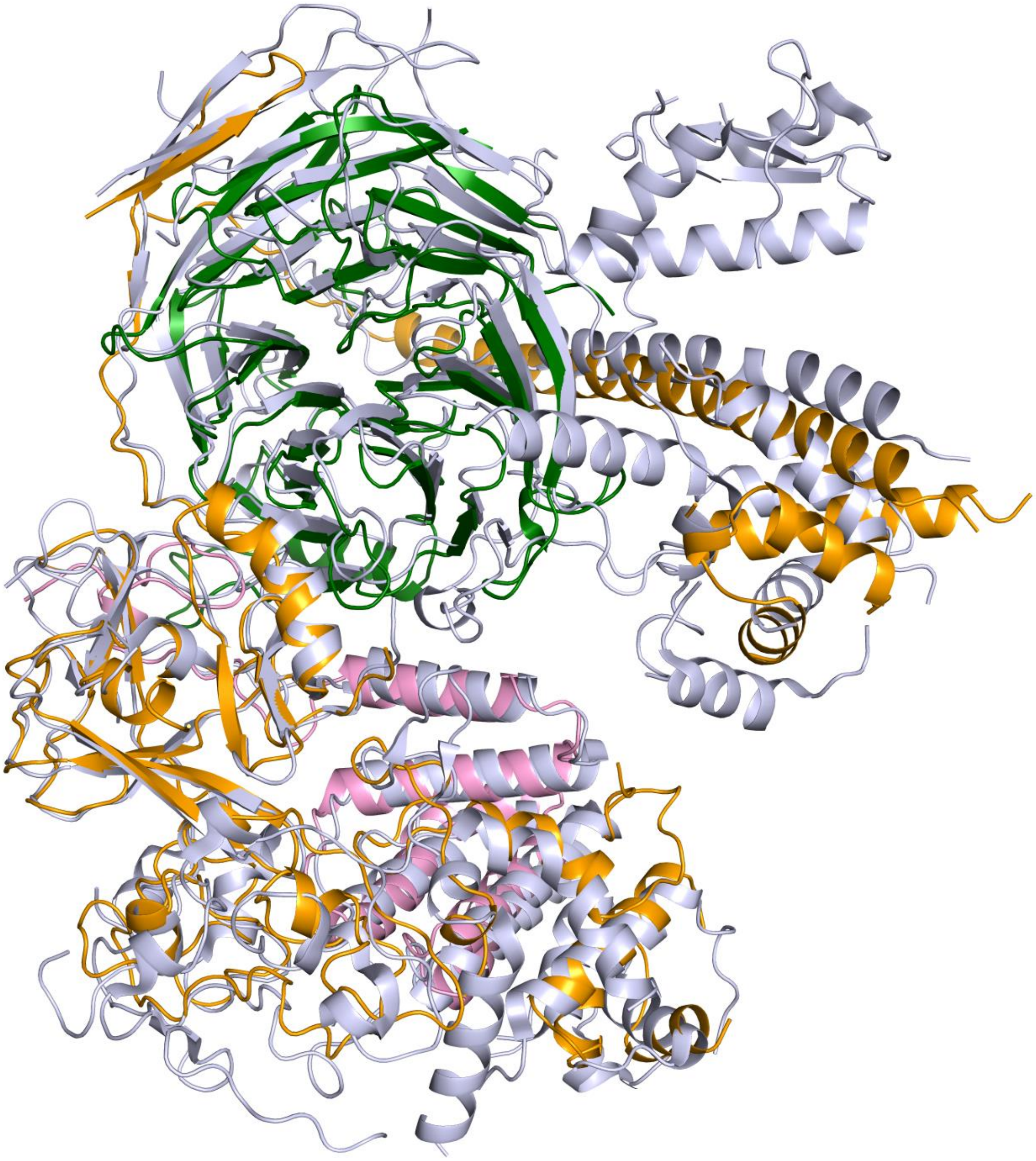

b

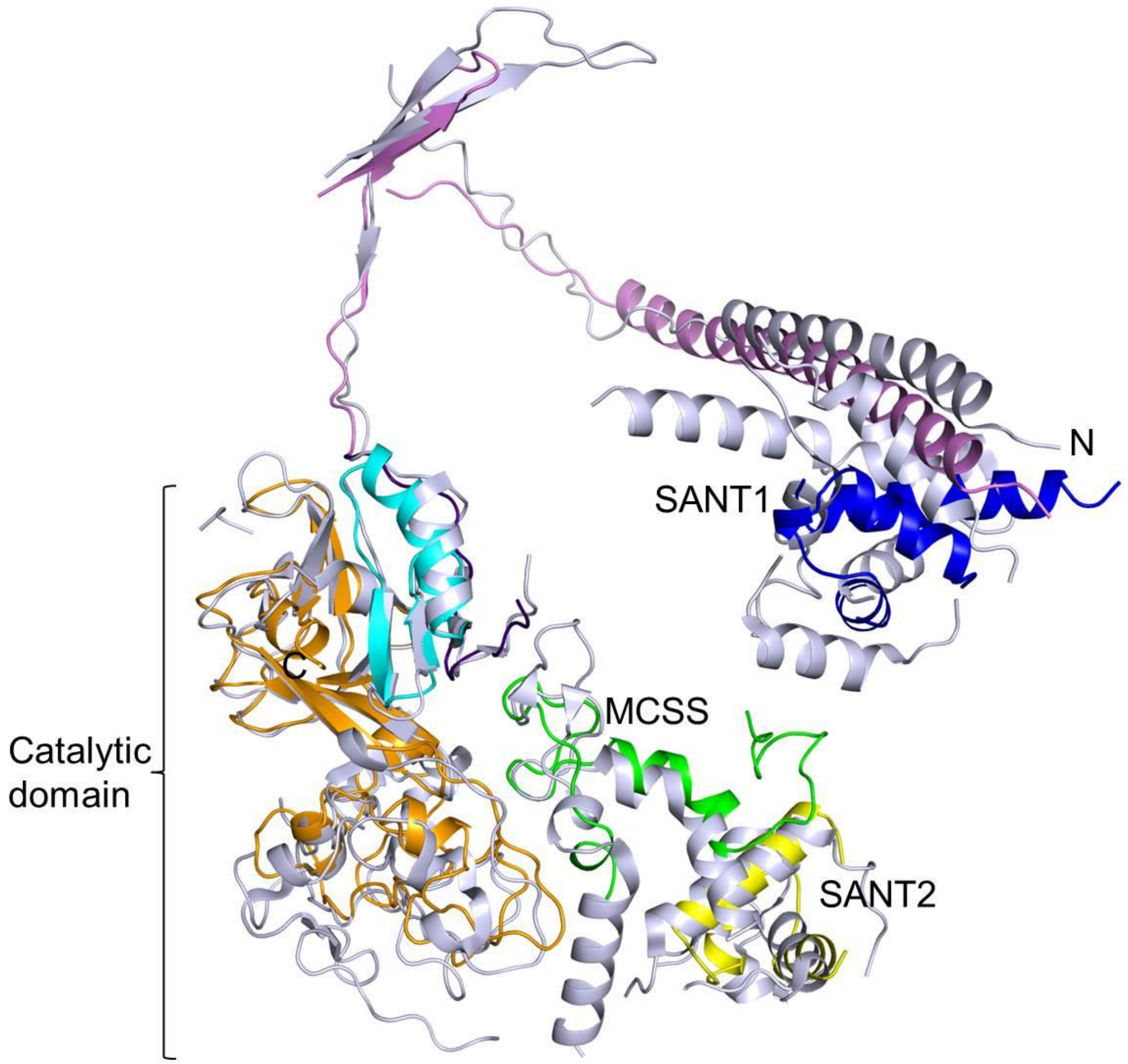

c

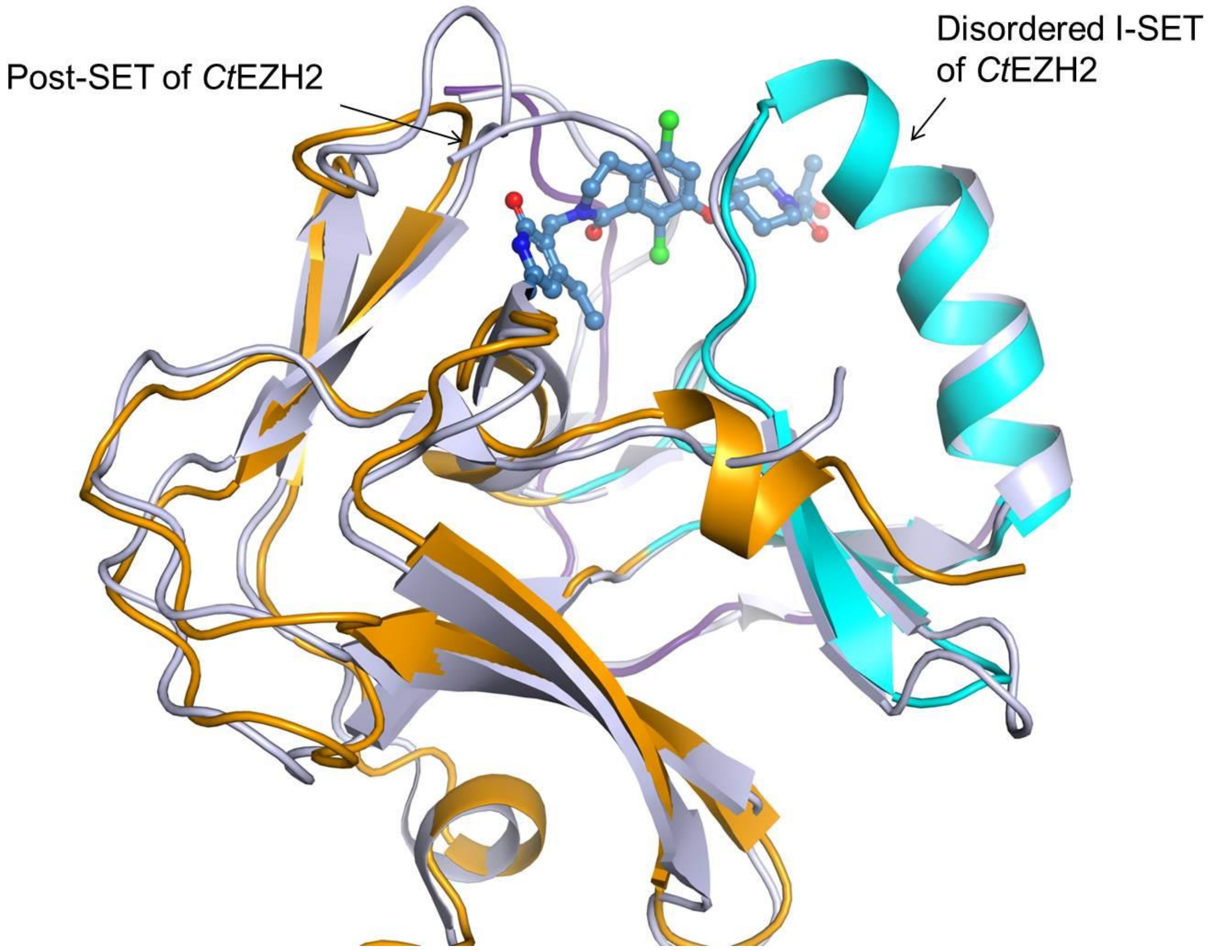

Supplementary Figure 3. Comparison of *Hs/Ac*PRC2 with *Ct*PRC2. *Ct*PRC2 is shown in gray. **a**, Overall architecture. Components of *Hs/Ac*PRC2 are shown in color: *Ac*EZH2_X in orange, *Hs*EED in green and *Hs*SUZ12 in pink. **b**, Comparison of the EZH2 subunits. Various domains of *Ac*PRC2_X are shown in the same color as in Fig. 3b. **c**, SET domain of *Ac*EZH2_X and *Ct*EZH2 highlighting key differences in the I-SET and post-SET regions. I-SET of *Ac*EZH2_X is highlighted in cyan.

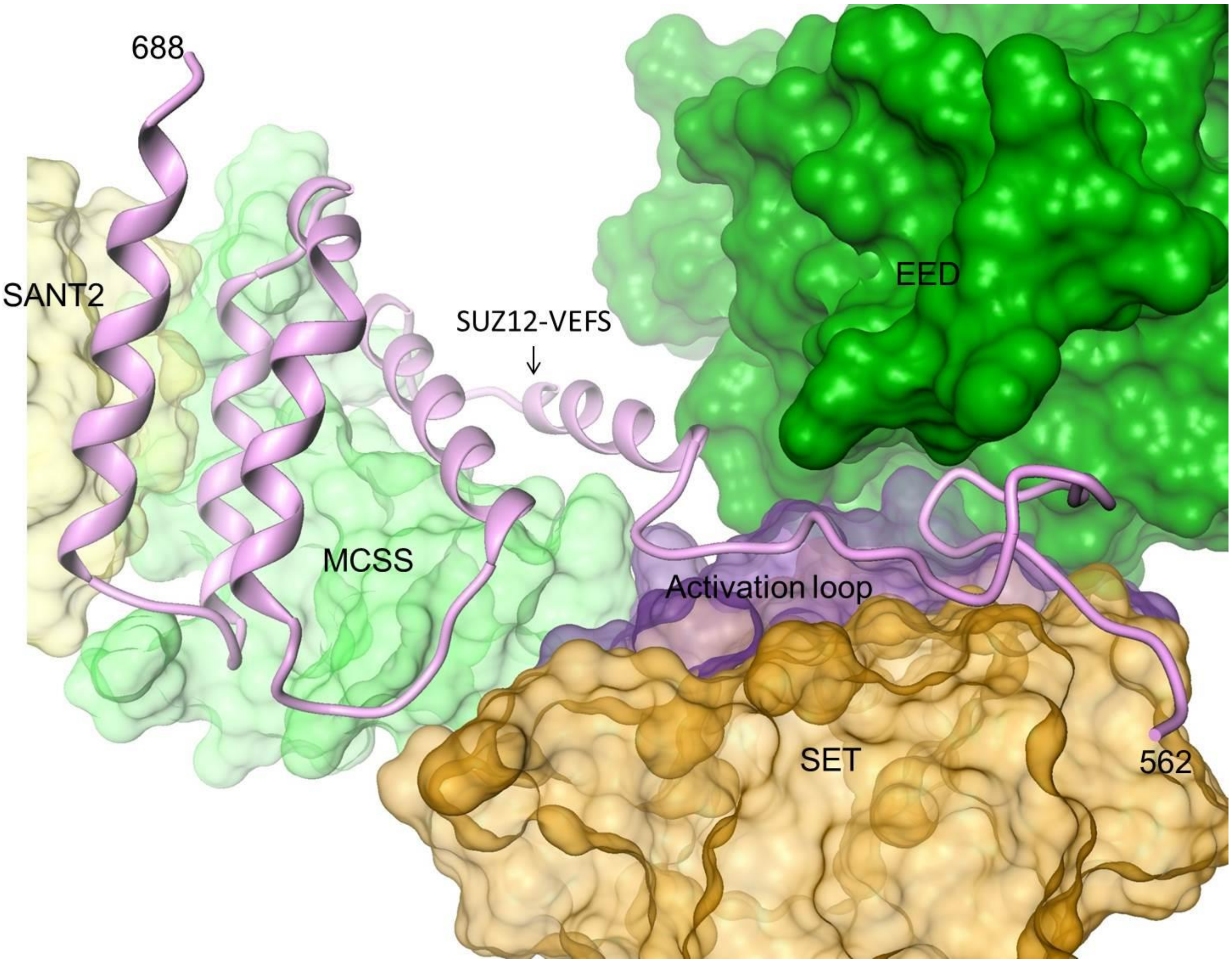

Supplementary Figure 4. SUZ12-VEFS (562 – 688) structure in the context of the complex. EED is shown in solid green surface on upper right; different parts of EZH2 interacting with SUZ12-VEFS are shown in transparent surfaces. The color code is the same as in Fig. 3: activation loop in indigo, MCSS in light green, SANT2 in yellow and SET domain is shown in orange.

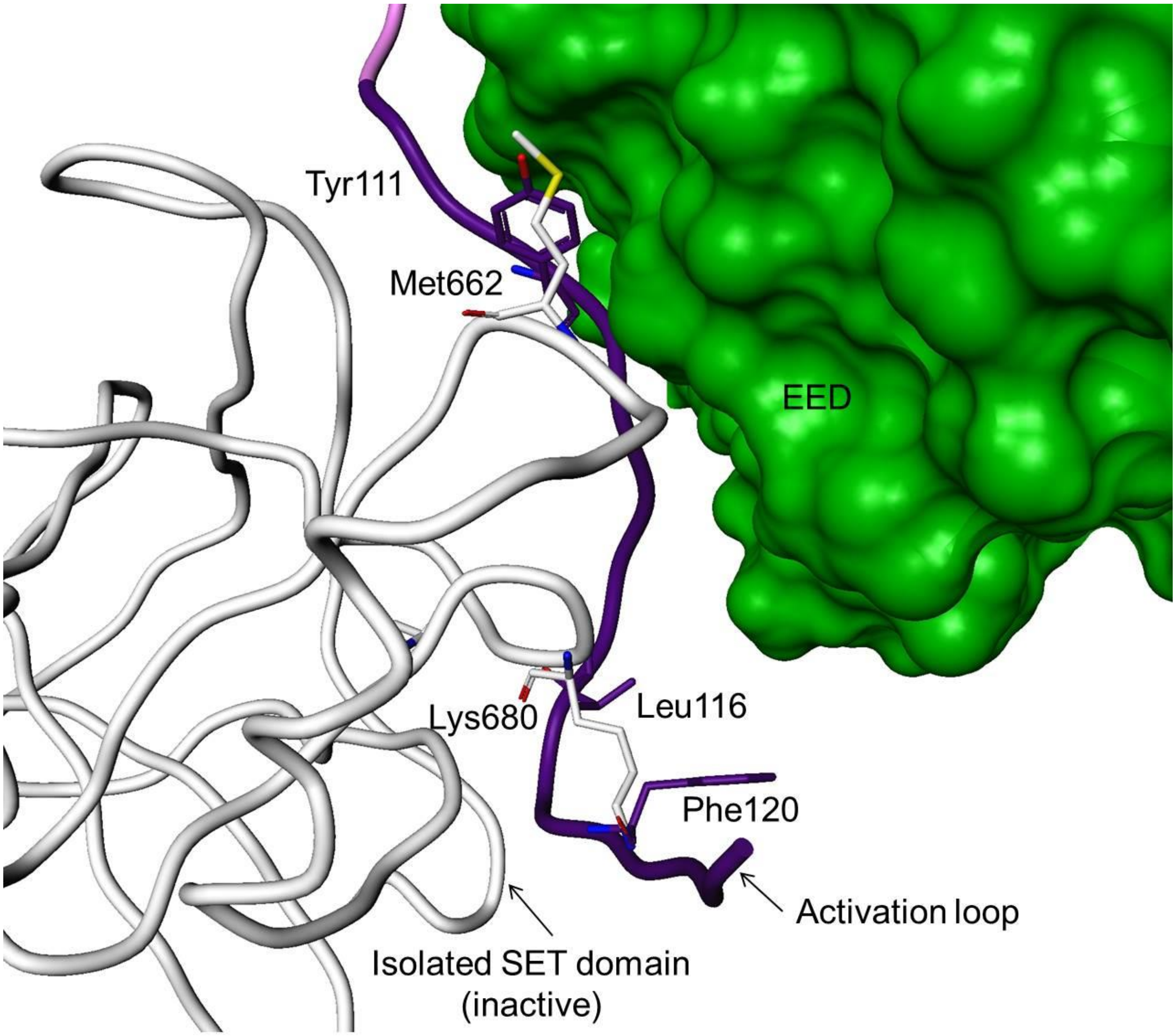

Supplementary Figure 5. PRC2 activation. When the SET domain of the PRC2 structure reported here is replaced with the inactive SET domain (PDBID: 4MI5), shown in gray, Met662 from the SET domain is in steric conflict with Tyr111 from the activation loop. Likewise, Lys680 from the inactive SET domain occupies the same space as Leu116 and Phe120 from the activation loop. Thus ordering of the activation loop must induce conformational change in the I-SET region to the catalytically competent state, stabilized via favorable interactions between the I-SET and the activation loop as shown in Fig. 5a.

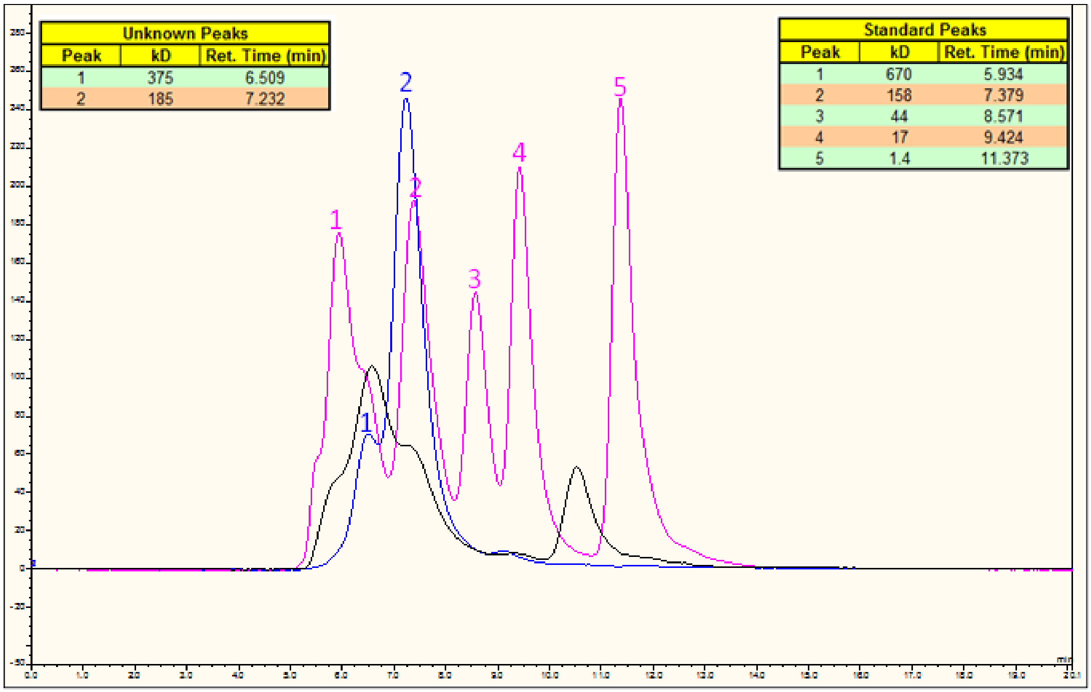

Supplementary Figure 6. Dimerization of Y641N *Hs/Ac*PRC2. aSEC profile of Y641N *Hs/Ac*PRC2 prior to preparative SEC (1.8 mg/ml black trace) with significant dimer fraction present, and purified monomer used for co-crystallization (9.5 mg/ml; blue trace)

a

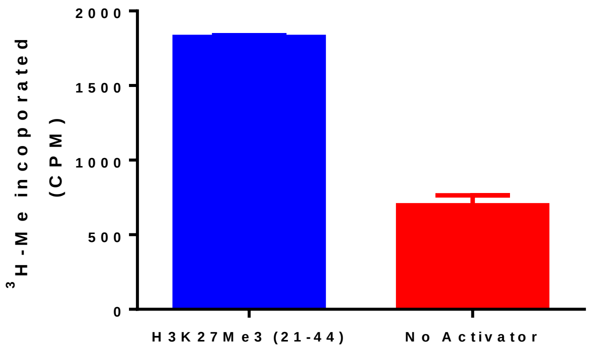

b

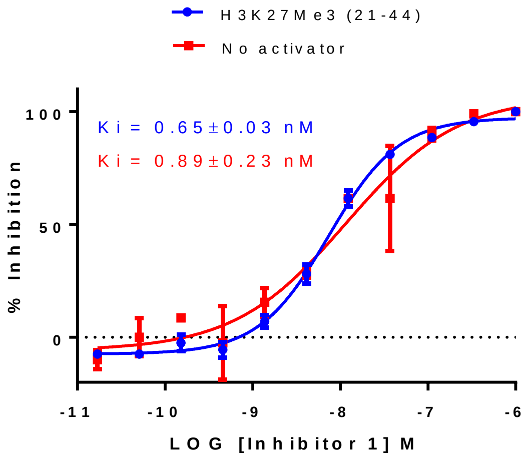

Supplementary Figure 7. Inhibition of basal and activated states of *Hs*PRC2_4 by compound 1. **a**, Activation of intact *Hs*PRC2 four-protein complex with 1.7 μM of activator peptide (H3K27Me3 21-44; 21st Century Biochemicals). K_a^{app}= 0.2 μM (data not shown) similar to K_a^{app}= 0.086 μM previously reported for *Hs*PRC2_4. **b**, Inhibition of intact PRC2 four protein complex by inhibitor 1 in the presence of the activator peptide (H3K27Me3 (21-44); 1.7 μM). The experiments were conducted at 15 μM SAM which is above $K_{m,}^{SAM}$ = 1.5 μM for intact *Hs*PRC2_4complex (mean of two independent experiments ± s.d).

a

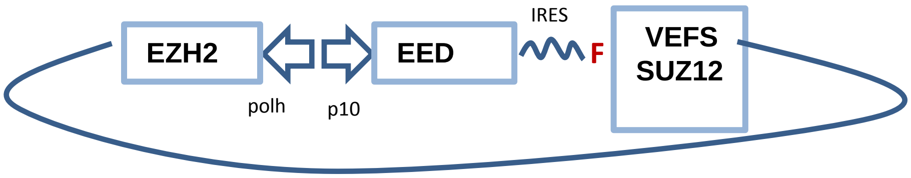

b

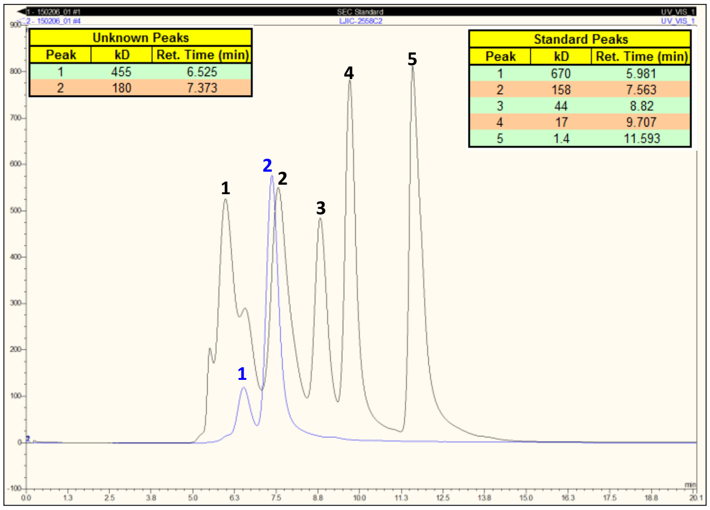

Supplementary Figure 8. Expression and aSEC characterization of PRC2. **a**, Schematic representation of the expression vector design to co-express subunits of PRC2 three protein complex (polH and p10 are respective promoters in pFASTBAC Dual vector; IRES is described in Methods section; F- Flag tag sequence). **b**, aSEC profile of *Hs/Ac*PRC2_X protein sample (17.8 mg/ml) used for co-crystallization with the compound 1.

^{1}H NMR ($CDCl_3$)

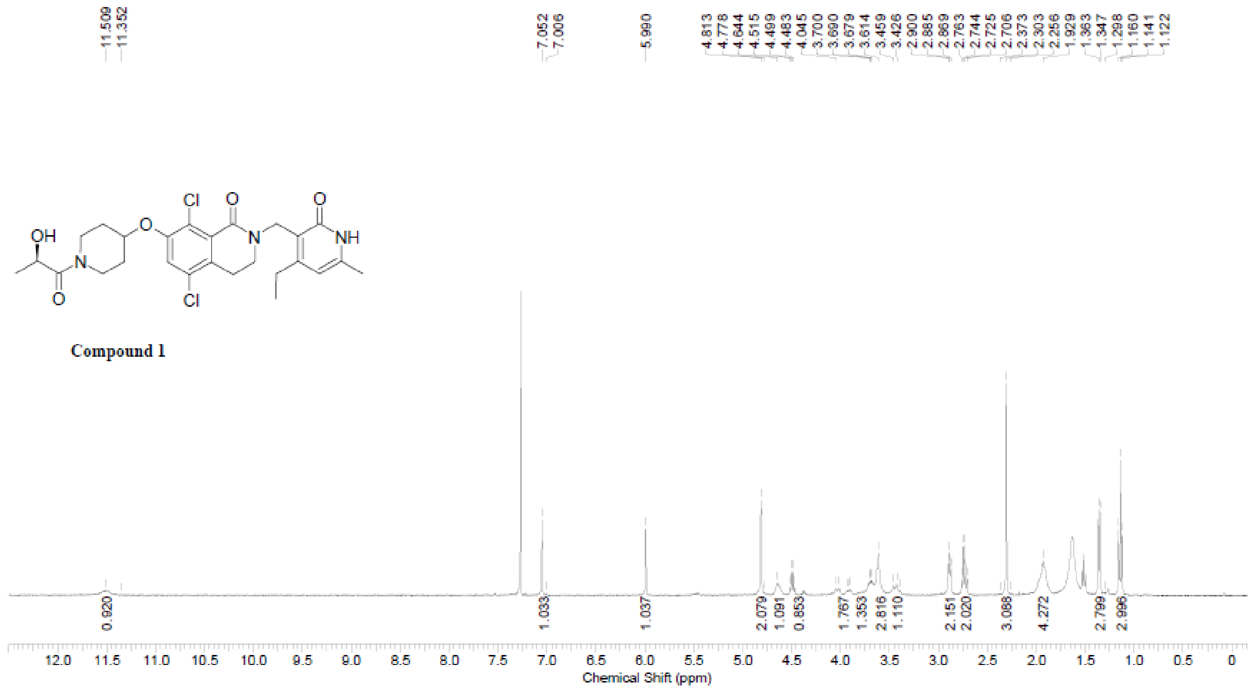

Compound 1

^{13}C NMR ($CDCl_3$)

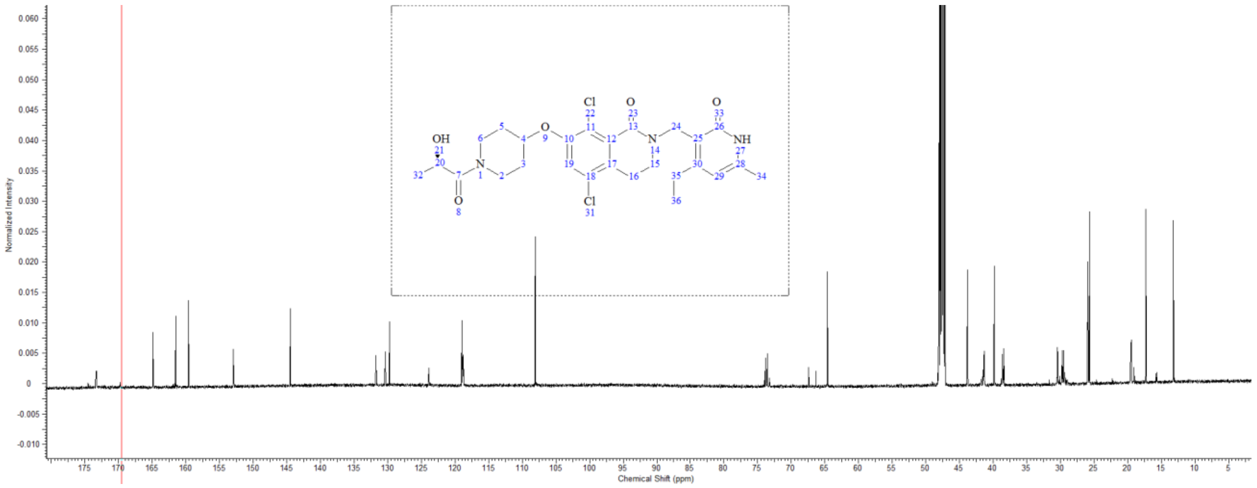

Supplementary Figure 9. NMR characterization of compound **1**.

Supplementary Table 1 SAXS analysis for subset of PRC2 complexes

Sample ID	Radius of Gyration (Å)	Mass (kDa)	Volume (nm^3)	Porod Exponent	Maximum Dimension (Å)
*Hs*PRC2	43.3	156	390	3.5	155
*Hs*PRC2 (Δ 342-419 EZH2)	45.3	147	390	3.4	300
*Hs/Ac*PRC2	44.7	175	416	3.6	168
*Hs/Ac*PRC2 (Δ 329-415 EZH2)	42.8	147	359	3.7	177

Supplementary Methods

Synthesis of compound 1: (*R*)-5,8-dichloro-2-((4-ethyl-6-methyl-2-oxo-1,2-dihydropyridin-3-yl)methyl)-7-((1-(2-hydroxypropanoyl)piperidin-4-yl)oxy)-3,4-dihydroisoquinolin-1(2H)-one

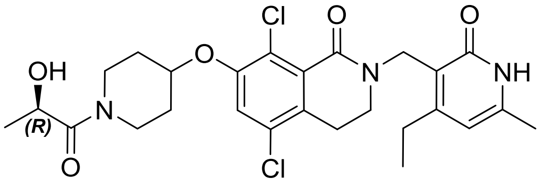

1

Synthesis of intermediates

Compound 296g: 5,8-dichloro-7-hydroxy-3,4-dihydroisoquinolin-1(2H)-one

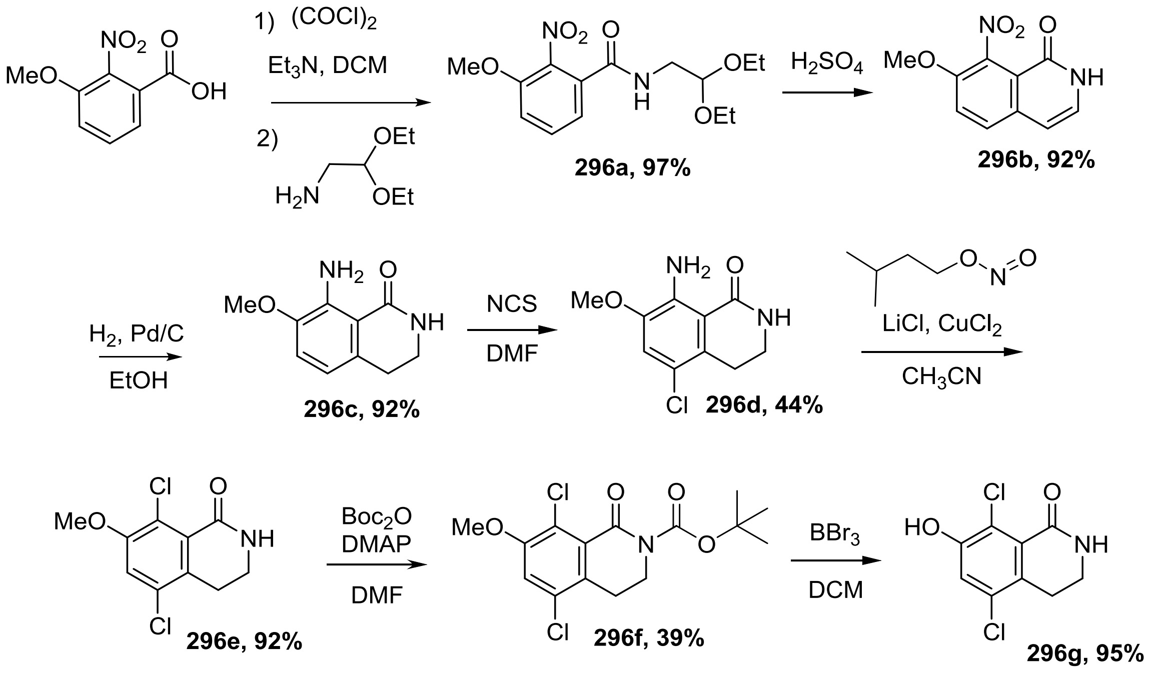

Oxalyl chloride (34 mL, 0.395 mol) was added to a solution of 3-methoxy-2-nitrobenzoic acid (60 g, 0.305 mol) in dry dichloromethane (600 mL), followed by N,N-dimethylformamide (0.6 mL, 7.8 mmol), which initiates mild gas evolution. The mixture was stirred at room temperature for two hours, then

concentrated under vacuum to remove volatiles. The crude acid chloride was dissolved in dry dichloromethane (150 mL) then added dropwise to a cooled (5 °C) solution of aminoacetaldehyde diethylacetal (48 mL, 0.33 mol) and triethylamine (52 mL, 0.37 mol) in dry dichloromethane (250 mL). The mixture was stirred at room temperature for two hours and then washed with saturated aqueous sodium bicarbonate (2 x 100 mL) and brine (100 mL). The organics were dried over sodium sulfate and concentrated to give *N*-(2,2-diethoxyethyl)-3-methoxy-2-nitrobenzamide (**296a**, 92 g, 97% yield) as a yellow solid. ^{1}H NMR (400 MHz, DMSO-d6) δ 8.83 (t, J=5.9 Hz, 1H), 7.63 (t, J=7.8 Hz, 1H), 7.47 (dd, J=0.8, 8.50 Hz, 1H), 7.25 (dd, J=0.9, 7.7 Hz, 1H), 4.56 (t, J=5.6 Hz, 1H), 3.90 (s, 3H), 3.62 (qd, J=7.0, 9.5 Hz, 2H), 3.49 (qd, J=7.0, 9.6 Hz, 2H), 3.25 (t, J=5.7 Hz, 2H), 1.12 (t, J=7.0 Hz, 6H); MS: 335 [M+Na$^+$].

A mixture of *N*-(2,2-diethoxyethyl)-3-methoxy-2-nitrobenzamide (**296a**, 92 g, 0.295 mol) in concentrated sulfuric acid (1 L) was stirred at 70 °C for three hours. After cooling to room temperature, the mixture was slowly poured into ice water (3 L), causing a solid precipitate to form. The precipitate was collected by filtration. The filter cake was washed with water (1 L) and dried to afford 7-methoxy-8-nitroisoquinolin-1(2*H*)-one (**296b**, 60 g, 92% yield) as a brown solid. ^{1}H NMR (400 MHz, DMSO-d6) δ 11.54 (br. s., 1H), 7.90 (d, J=8.9 Hz, 1H), 7.79 (d, J=9.0 Hz, 1H), 7.16 (t, J=6.4 Hz, 1H), 6.65 (d, J=7.1 Hz, 1H), 3.95 (s, 3H); MS: 221 [M+H].

A suspension of 7-methoxy-8-nitroisoquinolin-1(2*H*)-one (**296b**, 30 g, 0.136 mol) and 10% palladium on carbon (15 g, 0.014 mol) in ethanol (1 L) was stirred at 40 °C under hydrogen (20 psi) for 72 hours. The mixture was filtered through celite, the flask and filter pad washed with ethanol (1 L), and the combined filtrates concentrated under vacuum to give 8-amino-7-methoxy-3,4-dihydroisoquinolin-1(2*H*)-one (**296c**, 24 g, 92% yield) as a brown oil. ^{1}H NMR (400 MHz, DMSO-d6) δ 7.65 (br. s., 1H), 6.82 (d, J=7.8 Hz, 1H), 6.33 (d, J=7.8 Hz, 1H), 3.8 (s, 3H), 3.26 (dt, J=2.9, 6.5 Hz, 2H), 2.72 (t, J=6.5 Hz, 2H); MS: 193 [M+H].

N-chlorosuccinimide (20 g, 0.147 mol) was added to a solution of 8-amino-7-methoxy-3,4-dihydroisoquinolin-1(2*H*)-one (**296c**, 24 g, 0.125 mol) in N,N-dimethylformamide (250 mL) and stirred at room temperature overnight. The solution was partitioned between water (100 mL) and ethyl acetate (100 mL). The aqueous layer was further extracted with ethyl acetate (4 x 100 mL). The combined organic extracts were washed with brine (5 x 100 mL), dried over sodium sulfate, and concentrated to dryness. The residue was triturated with acetonitrile (200 mL), and the solids collected by filtration. After drying, 8-amino-5-chloro-7-methoxy-3,4-dihydroisoquinolin-1(2*H*)-one (**296d**, 12.5 g, 44% yield)

was obtained as a blue solid. ^{1}H NMR (400 MHz, DMSO-d6): δ 7.84 (s, 1H), 6.93 (s, 1H), 3.80 (s, 3H), 3.29-3.25 (m, 2H), 2.81-2.78 (t, J= 6.6 Hz, 2H); MS: 246 [M+H]

Isopentyl nitrite (20 mL, 0.15 mol) was added dropwise to a heated (55 °C) suspension of copper (II) chloride (40 g, 0.30 mol) and lithium chloride (38 g, 0.91 mol) in acetonitrile (500 mL). The mixture was stirred at that temperature for 5 minutes. Then, 8-amino-5-chloro-7-methoxy-3,4-dihydroisoquinolin-1(2*H*)-one (**296d**, 20 g, 0.089 mol) was added in 3 – 5 g portions. After the addition was complete, stirring was continued at 55 °C for 45 minutes. The reaction mixture was cooled to room temperature, quenched with saturated aqueous ammonium chloride (300 mL), and extracted with ethyl acetate (4 x 200 mL). The combined organic layers were washed with aqueous ammonium chloride (200 mL) and brine (100 mL). After drying over sodium sulfate, the extract was concentrated to give 5,8-dichloro-7-methoxy-3,4-dihydroisoquinolin-1(2*H*)-one (**296e**, 20 g, 90% purity, 92% yield) as a brown solid. ^{1}H NMR (400 MHz. DMSO-d6): δ 8.26 (br s, 1H), 7.40 (s, 1H), 3.09 (s, 3H), 3.29-3.25 (m, 2H), 2.88-2.85 (t, J= 6.6 Hz, 2H); MS: 246 [M+H].

Di-*tert*-butyl dicarbonate (76 g, 0.352 mol) was added in portions to a cooled (0 °C) solution of crude 5,8-dichloro-7-methoxy-3,4-dihydroisoquinolin-1(2*H*)-one (**296e**, 20 g, 0.082 mol) and 4-dimethylaminopyridine (30 g, 0.246 mol) in N,N-dimethylformamide (200 mL). After the addition was complete, the solution was stirred at room temperature overnight. The reaction mixture was partitioned between water (200 mL) and ethyl acetate. The aqueous layer was further extracted with ethyl acetate (4 x 200 mL). The combined organic extracts were dried over sodium sulfate, concentrated, and purified by silica gel chromatography (eluting with petroleum ether/ethyl acetate 100:1 to 10:1) to give *tert*-butyl 5,8-dichloro-7-methoxy-1-oxo-3,4-dihydroisoquinoline-2(1*H*)-carboxylate (**296f**, 11 g, 39% yield) as a yellow solid. ^{1}H NMR (400 MHz, DMSO-d6): δ 7.50 (s, 1H), 4.00 (s, 3H), 3.86-3.83 (t, J= 6.8 Hz, 2H), 2.99-2.96 (t, J= 5.8 Hz, 2H), 1.54 (s, 9H).

Neat boron tribromide (26 g, 105 mmol) was added to a cooled (0 °C) solution of *tert*-butyl 5,8-dichloro-7-methoxy-1-oxo-3,4-dihydroisoquinoline-2(1*H*)-carboxylate (**296f**, 14.5 g, 45.4 mmol) in dry dichloromethane (100 mL). The mixture was stirred at room temperature overnight. Water (10 mL) was added causing a precipitate to form. The precipitate was collected by filtration, washed with water (500 mL), and dried to give 5,8-dichloro-7-hydroxy-3,4-dihydroisoquinolin-1(2*H*)-one (**296g**, 9.2 g, 95% yield) as a yellow solid. ^{1}H NMR (400 MHz, DMSO-d6): δ 10.58 (s, 1H), 8.17 (s, 1H), 7.13 (s, 1H), 3.25-3.23 (m, 2H), 2.83-2.80 (t, J=6.2 Hz, 2H). MS: 232 [M+H].

Compound EE: 2-(benzyloxy)-3-(chloromethyl)-4-ethyl-6-methylpyridine

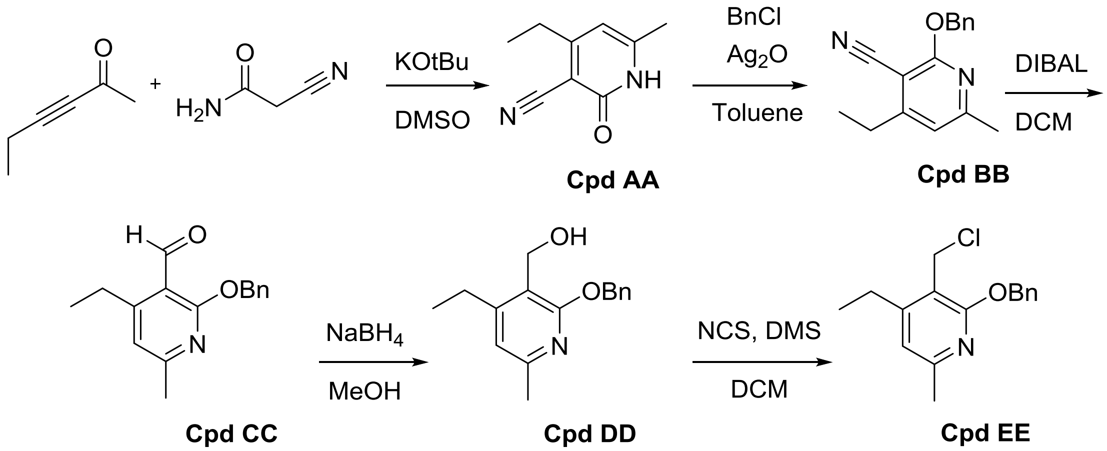

A solution of 2-cyano-acetamide (841 mg, 10.0 mmol) in DMSO (20 mL) and potassium *tert*-butoxide (1.18 g, 10.5 mmol) was stirred at 23 °C for 30 minutes. The mixture was cooled to 0 °C and then 3-hexyn-2-one (1.1 mL, 10 mmol) was added. The reaction mixture was stirred for 2 hours at 0 °C. The reaction mixture was quenched with saturated aqueous ammonium chloride (3 mL) and diluted further with water (10 mL) causing a solid to precipitate out. The suspension was filtered and the solids dried under vacuum to give 4-ethyl-6-methyl-2-oxo-1,2-dihydropyridine-3-carbonitrile (**Cpd AA**, 1.2 g, 71%) as a white solid. ^{1}H NMR (400 MHz, DMSO-d6): δ 1.17 (t, J=7.6 Hz, 3 H) 2.25 (s, 3 H) 2.54 - 2.67 (m, 2 H) 6.22 (s, 1 H); MS: 163 [M+H].

A mixture of 4-ethyl-6-methyl-2-oxo-1,2-dihydropyridine-3-carbonitrile (**Cpd AA**, 1.1 g, 6.8 mmol), (chloromethyl)benzene (1.1 mL, 9.4 mmol) and silver(I) oxide (1.8 g, 7.7 mmol) in anhydrous toluene (23 mL) was heated at 110 °C for 17 hours. The reaction mixture was cooled to 23 °C then filtered through CELITE®. The filtrate was concentrated under vacuum. The residue was purified by column chromatography (heptane/ EtOAc) to give 2-(benzyloxy)-4-ethyl-6-methylpyridine-3-carbonitrile (**Cpd BB,** 1.42 g, 83%) as a colorless oil. ^{1}H NMR (CHLOROFORM-d): δ 7.51 (d, J=7.3 Hz, 2H), 7.29-7.43 (m, 3H), 6.72 (s, 1H), 5.50 (s, 2H), 2.78 (q, J=7.6 Hz, 2H), 2.48 (s, 3H), 1.28 (t,J=7.6 Hz, 3H); MS: 253 [M+H].

To a -5 °C solution of 2-(benzyloxy)-4-ethyl-6-methylpyridine-3-carbonitrile (**Cpd BB**, 0.687 g, 2.72 mmol) in dichloromethane (9 mL) was added diisobutylalluminum hydride (3 mL of a 1M solution in DCM, 3 mmol). After 3 hours, the reaction mixture was quenched with 1M aqueous hydrochloric acid (3 mL). After stirring for 15 minutes, a 2M aqueous solution of potassium sodium tartrate (Rochelle's salt) (3 mL) was added. After stirring for 30 min., the resulting mixture was filtered through CELITE®. The

filtrate was concentrated under vacuum to remove the DCM and the water layer was extracted with ethyl acetate (3 x 40 mL). The combined organic extract was washed with brine (10 mL), dried over sodium sulfate, filtered, and concentrated. The resulting residue was purified by column chromatography (heptane/ EtOAc) to give 2-(benzyloxy)-4-ethyl-6-methylpyridine-3-carbaldehyde (**Cpd CC,** 323 mg, 46%) as a colorless oil. ^{1}H NMR (CHLOROFORM-d): δ 10.57 (s, 1H), 7.48 (d, J=7.3 Hz, 2H), 7.30-7.43 (m, 3H), 6.69 (s, 1H), 5.50 (s, 2H), 2.99 (q, J=7.6 Hz, 2H), 2.48 (s, 3H), 1.20 (t, J=7.5 Hz, 3H); MS: 256 [M+H].

To a 0 °C solution of 2-(benzyloxy)-4-ethyl-6-methylpyridine-3-carbaldehyde (**Cpd CC**, 323 mg, 1.28 mmol) in methanol (4.27 mL) was added sodium borohydride (54 mg, 1.41 mmol). After 1 hour, the reaction mixture was concentrated and diluted with ethyl acetate (20 mL) and water (10 mL). The organic layer was washed with brine (5 mL), dried over sodium sulfate, filtered, and concentrated under vacuum. The residue was purified by column chromatography (heptane/ EtOAc) to give [2-(benzyloxy)-4-ethyl-6-methylpyridin-3-yl]methanol (**Cpd DD**, 280 mg, 85% yield) as a colorless oil. ^{1}H NMR (CHLOROFORM-d): δ 7.44-7.51 (m, 2H), 7.29-7.43 (m, 3H), 6.63 (s, 1H), 5.44 (s, 2H), 4.70 (d, J=3.9 Hz, 2H), 2.67 (q, J=7.6 Hz, 2H), 2.43 (s, 3H), 2.34 (br. s., 1H), 1.21 (t, J=7.6 Hz, 3H); MS: 258 [M+H].

To a 0 °C solution of N-chlorosuccinimide (81.5 mg, 0.598 mmol) in dichloromethane (2.47 mL) was added dimethyl sulfide (48 μl, 0.653 mmol). The reaction mixture was then cooled to -20 °C and a solution of [2-(benzyloxy)-4-ethyl-6-methylpyridin-3-yl]methanol (**Cpd DD**, 140 mg, 0.554 mmol) in dichloromethane (1 mL) was added dropwise. After 2 hours, the reaction mixture was poured into brine (5 mL) and the aqueous layer extracted with dichloromethane (2 x 10 mL). The combined organic extracts were dried over sodium sulfate, filtered, and concentrated. The crude product was purified by column chromatography (heptane/ EtOAc) to give the title compound (**Cpd EE**, 35 mg, 23% yield) as a colorless oil. ^{1}H NMR (CHLOROFORM-d): δ 7.52 (d, J=7.3 Hz, 2H), 7.29-7.44 (m, 3H), 6.65 (s, 1H), 5.46 (s, 2H), 4.74 (s, 2H), 2.72 (q, J=7.6 Hz, 2H), 2.44 (s, 3H), 1.28 (t, J=7.6 Hz, 3H).

Synthesis of 1:

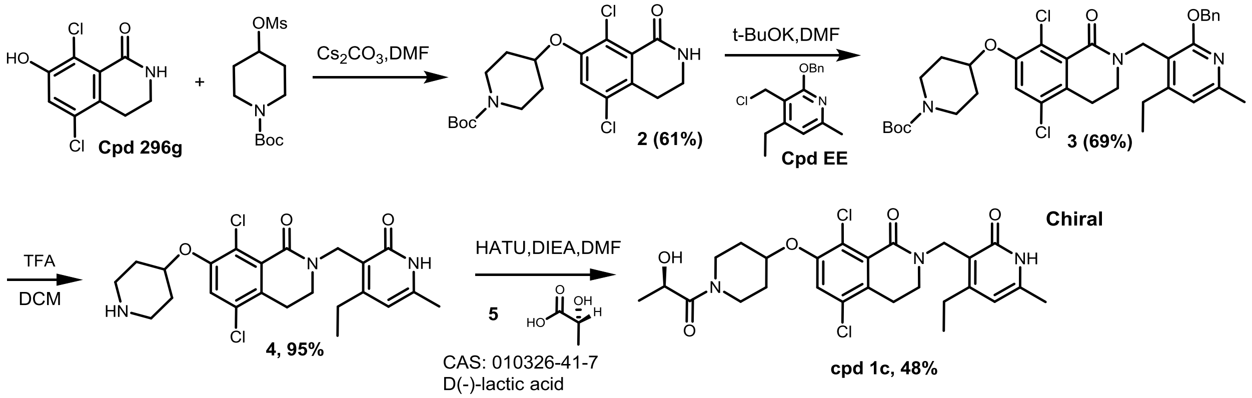

A suspension of **296g** (200 mg, 0.862 mmol), tert-butyl 4-((methylsulfonyl)oxy)piperidine-1-carboxylate (270 mg, 0.967 mmol) and Cs_2CO_3 (850 mg, 5.17 mmol) in dry DMF (10 mL) was heated at 80 °C for 18 h. The reaction mixture became yellow over this time. TLC (petroleum ether: EtOAc= 1: 1, Rf ~ 0.35) showed **296g** was consumed and a new spot was detected. To the mixture was added H_2O (10 mL) and the organics were extracted with EtOAc (3 x 10 mL). The combined organic layers were washed with brine (3 x 10 mL), dried over Na_2SO_4, and concentrated to give the crude product as yellow oil. The crude product was purified by flash chromatography (silica gel, DCM: MeOH= 15: 1) to give compound **2** (220 mg, 61%) as a yellow solid. ^{1}H NMR (400 MHz, CHLOROFORM-d): δ 7.11 (s, 1 H), 6.07 (br. s., 1 H), 4.53 (m, 1 H), 3.61 - 3.72 (m, 2 H), 3.41 - 3.52 (m, 4 H), 3.03 (t, J=6.36 Hz, 2 H), 1.79 - 1.96 (m, 4 H), 1.48 (s, 9 H); MS: 359 / 361 [M –tBu].

To a mixture of compound **2** (220 mg, 0.530 mmol) in dry DMF (4.0 mL) was added KOtBu (0.6 mL, 0.6 mmol, 1M in THF) at 0 °C under N_2. After stirring for 5 min., compound **EE** (140 mg, 0.508 mmol) was added in one portion. Then, the mixture was stirred at 0 °C for 10 min. TLC (petroleum ether : EtOAc = 3 : 1, Rf ~0.4) showed that compound **EE** had been consumed. To the reaction mixture was added 2 drops of AcOH at 0 °C. The temperature increased from 0 °C to 10 °C during the addition. The mixture was diluted with MTBE (50 mL). The organic extract was washed with brine (2 x 25 mL), dried over Na_2SO_4 and concentrated to afford 400 mg of the crude product as a yellow oil. This material was purified by flash chromatography (silica gel, petroleum ether: EtOAc =3: 1) to give compound **3** (230 mg, 69 %) as a yellow gum. ^{1}H NMR (400 MHz, CHLOROFORM-d): δ ppm 7.42 - 7.48 (m, 2 H), 7.29 - 7.39 (m, 3 H), 7.03 (s, 1 H), 6.63 (s, 1 H), 5.43 (s, 2 H), 4.88 (s, 2 H), 4.49 (tt, J=6.3, 3.3 Hz, 1 H), 3.62 - 3.71 (m, 2 H),

3.40 - 3.49 (m, 2 H), 3.26 (t, J=6.2 Hz, 2 H), 2.73 (q, J= 7.6 Hz, 2H), 2.69 (t, J=6.2 Hz, 2 H), 2.33 (s, 3 H), 1.78 - 1.94 (m, 4 H), 1.48 (s, 9 H), 1.14 (t, J= 7.6 Hz, 3H); MS: 654/656 [M+H].

To a solution of compound **3** (230 mg, 0.351 mmol) in dry DCM (5 mL) was added TFA (5 mL) dropwise at 0 ^{o}C. After the addition, the mixture was stirred at 25 ^{o}C for 18 hours. The reaction mixture became yellow. The pH of the mixture was adjusted to pH ~9 using sat. aq. $NaHCO_3$ and then the mixture was diluted with DCM (30 mL). The aqueous layer was extracted with DCM (3 x 30 mL) and the combined organic layers were washed with brine (30 mL), dried over Na_2SO_4 and concentrated to give the crude product as a yellow oil. This material was dissolved in MeOH (30 mL) and solid K_2CO_3 (200 mg, 1.45 mmol) was added. The yellow suspension was stirred at 25 ^{o}C for 15 min. The mixture was filtered through a pad of Celite and the filter cake was washed with DCM (20 mL). The filtrate was concentrated to give the crude product as a yellow gum. The product was purified by flash chromatography (DCM : MeOH = 10 : 1, Rf ~ 0.2). The resulting product was suspended in water, frozen and lyophilized for 18 hours to obtain compound **4** (155 mg, 95%) as an off-white solid. MS: 450 / 452 [M+H].

To a stirred solution of compound **4** (155 mg, 0.334 mmol) in dry DMF (10 mL), was added compound **5** (35.0 mg, 0.389 mmol), DIEA (150 mg, 1.16 mmol) and HATU (165 mg, 0.434 mmol) at 0 ^{o}C under N_2. The mixture was warmed to 25 ^{o}C and stirred at 25 ^{o}C for 30 min. The reaction was cooled to 0 ^{o}C and quenched with H_2O (10 mL). The organics were extracted with EtOAc (3 x 30 mL) and the combined organic extract was washed with sat. aq. Na_2CO_3 (4 x 30 mL), H_2O (30 mL) and brine (2 x 30 mL). After drying over Na_2SO_4, concentration of the extract gave the crude product as a yellow gum. The crude product was purified by preparative TLC (silica gel, EtOAc: MeOH=15: 1, Rf ~0.3) to afford the product which was suspended in water, frozen and lyophilized for 18 hours to give compound **1** (85 mg, 48%) as a white solid. ^{1}H NMR (400 MHz, CDCl3): δ 11.35 (brs, 1H), 7.05 (s, 1H), 5.99 (s, 1H), 4.81 (s, 2H), 4.77-4.64 (m, 1H), 4.52-4.48 (m, 1H), 4.05-3.70 (m, 2H), 3.69-3.68 (m, 1H), 3.67-3.61 (m, 3H), 3.46-3.43 (m, 1H), 2.88 (t, J= 5.8 Hz, 2H), 2.73 (q, J= 7.6 Hz, 2H), 2.30 (s, 3H), 1.95-1.91 (m, 4H), 1.35 (d, J= 6.4 Hz, 3H), 1.14 (t, J= 7.6 Hz, 3H). ^{13}C NMR (151 MHz, MeOD) δ 174.78, 166.31, 162.95, 161.03, 154.36, 145.91, 133.15, 131.84, 131.17, 125.33, 120.41, 109.51, 75.29, 68.86, 45.26, 42.85, 41.28, 40.08, 31.94, 31.18, 27.35, 21.01, 18.75, 14.66. (Supplementary Fig. 9)

HRMS (m/z): [M]+ calcd for C26H31Cl2N3O5 = 535.1641; found 535.1641

Chiral purity = 100% via chiral SFC using the opposite enantiomer as a standard.

Column: Chiralpak AD-3 150×4.6mm I.D., 3um; Retention Time: 14.05 min

Mobile phase: 35% methanol (0.1% ethanolamine) in CO_2 flow rate: 2.4 mL/min. Wavelength: 220 nm.

References

1 Robert, X. & Gouet, P. Deciphering key features in protein structures with the new ENDscript server. *Nucleic acids research* **42**, W320-324, doi:10.1093/nar/gku316 (2014).
